# Self-Reported Medication Adherence Measured With Morisky Medication Adherence Scales and Its Determinants in Hypertensive Patients Aged ≥60 Years: A Systematic Review and Meta-Analysis

**DOI:** 10.3389/fphar.2019.00168

**Published:** 2019-03-01

**Authors:** Bartosz Uchmanowicz, Ewa A. Jankowska, Izabella Uchmanowicz, Donald E. Morisky

**Affiliations:** ^1^Department of Clinical Nursing, Faculty of Health Sciences, Wroclaw Medical University, Wroclaw, Poland; ^2^Cardiology Department, Centre for Heart Diseases, Military Hospital, Wroclaw, Poland; ^3^Department of Community Health Sciences, UCLA Fielding School of Public Health, Los Angeles, CA, United States

**Keywords:** hypertension, elderly patients, medication adherence, Morisky Medication Adherence Scale, Morisky Green Levine Medication Adherence Scale

## Abstract

**Background:** The aim of this systematic review and meta-analysis was to estimate medication adherence in hypertensive patients aged ≥60 years and to explore potential determinants of adherence with antihypertensive treatment in this age group.

**Methods:** A systematic search of the PubMed, Scopus, and Google Scholar using the Cochrane guidelines was performed. The analysis included articles published between 1 January 2000 and 30 June 2018. The patients were considered adherent if they scored ≥6 pts. on the Morisky Medication Adherence Scale (MMAS-8) or ≥3 pts. on the Morisky Green Levine Medication Adherence Scale (MGL). If available, also odds ratios (OR) with 95% confidence intervals (95% CI) for determinants of medication adherence were recorded.

**Results:** Thirteen studies including a total of 5,247 patients were available for the meta-analysis. The pooled percentage of adherence was 68.86% (95% CI: 57.80–79.92%). Subgroup analysis did not demonstrate a significant difference in the adherence measured with the MMAS-8 and the MGL (68.31 vs. 70.39%, *P* = 0.773). The adherence of patients from Western countries (Europe, United States) turned out to be significantly higher than in other patients (83.87 vs. 54.30%, *P* = 0.004). The significant determinants of better adherence identified in more than one study were older age, retirement/unemployment, duration of hypertension >10 years, and a lower number of prescribed drugs.

**Conclusion:** Medication adherence in the oldest old hypertensive patients seems to be higher than in younger persons. Adherence in older persons was associated with age, socioeconomic status, and therapy-related factors.

## Introduction

Despite considerable progress in the diagnosis and treatment of arterial hypertension, this condition still constitutes a serious medical, social, and economic burden. Blood pressure control, the primary objective of antihypertensive treatment, cannot be achieved even with the most efficacious medications but without cooperation on the patient's side. However, available evidence suggests that even up to 90% of hypertensive patients may not adhere to their therapies, and ~50% of them may discontinue the treatment within a year from the diagnosis (He et al., 2016).

A number of previous studies (Alhaddad et al., 2016; Cummings et al., 2016; Li et al., 2016) demonstrated that medication adherence in hypertensive patients increases with chronological age. However, the adherence to antihypertensive treatment is not a simple function of age but may also be modulated by a number of patient-related characteristics, such as functional limitations and cognitive impairment (Jankowska-Polańska et al., 2016). Indeed, some studies showed an inverse correlation between the age and adherence to antihypertensive medications (Jackevicius et al., 2002; Lam et al., 2007). Thus, a question arises whether the elderly, whose mental and physical performance are often deteriorated (Jankowska-Polańska et al., 2016), are truly more adherent than the younger patients. To the best of our knowledge, this issue has not been addressed adequately by previous studies and, as shown further in this paper, only a few of them analyzed medication adherence specifically in hypertensive patients aged 60 years and older, i.e., at the age when a person is generally considered “elderly.”

The adherence can be estimated either directly, based on the drug or its metabolite concentration in blood and urine, presence of a biological marker delivered with the drug and observation of patient's medication-taking behavior, or indirectly, with various questionnaires. While definitively less accurate than the direct assessment, questionnaire studies with validated scales are easier to conduct and hence, more suitable for the examination of larger patient populations in a community setting (Nielsen et al., 2017). Among a few available medication adherence scales, the most widespread is Morisky Medication Adherence Scale (MMAS) (Morisky et al., 1986). Originally designed as a four-item scale, the Morisky, Green and Levine (MGL) Medication Adherence Scale, with “yes” or “no” response categories, since 2008 it is also available in an eight-item version (MMAS-8), which aside from seven dichotomous statements, includes also one scored on a five-point Likert-type scale (Morisky et al., 2008). Irrespective of the MMAS type, the results are interpreted the same, i.e., the higher the score, the most likely are the respondents adherent to their treatment. Typically, the patient is considered adherent when his/her score is at least 3 pts. for the MGL Medication Adherence Scale or at least 6 pts. for the MMAS-8 scale, which corresponds from moderate to high adherence (Morisky et al., 1986, 2008). However, some authors use maximum values of the scales, 4 pts. for the MGL Medication Adherence Scale, and 8 pts. for the MMAS-8 as a medication adherence criterion (Lo et al., 2016; Bandi et al., 2017; Son and Won, 2017), which may be a source of bias in systematic reviews and comparative analyses. Nevertheless, due to their outstanding validity and reliability in patients with arterial hypertension and other chronic conditions, both Morisky Medication Adherence Scales are the most accepted self-reported medication adherence measures, recommended to serve as screening tools in a clinical setting. Furthermore, these scales were shown to correlate strongly with many fiscally important long-term outcomes, such as sustained behavioral change for individuals who receive educational counseling, proportion of treated patients compared to those having their health condition under control, frequency of emergency department visits, 30-day hospital readmission rate, morbidity, and mortality (Lam and Fresco, 2015).

The aim of this systematic review and meta-analysis was to estimate the percentage of hypertensive patients aged 60 years and older who are adherent to their treatment and to explore potential determinants of antihypertensive medication adherence/non-adherence in this age group.

## Methods

### Search Strategy

A systematic search of the PubMed, Scopus, and Google Scholar using the Cochrane guidelines to conduct the meta-analysis following PRISMA (Preferred Reporting Items for Systematic review and Meta-Analysis) statement was used. All published studies that addressed the issue of antihypertensive medication adherence and used the term “hypertension” as the MeSH major topic AND “adherence” OR “compliance” AND “Morisky medication adherence scale” OR “MMAS” OR “Morisky Green Levine medication adherence scale” OR “MGL medication adherence scale” MeSH subheadings were identified. The search limits were defined as “English” (language), “1 January 2000” and “30 June 2018” (publication date), and “humans” (species). Similar to Nielsen et al. (2017), we excluded studies conducted before 2000, to include hypertension definitions revised by the WHO in 1999 (Chalmers et al., 1999). The review was limited to self-reported studies that were conducted using the MMAS-8 or the MGL Medication Adherence Scale validated questionnaires administered to the hypertensive patients using antihypertensive medications.

### Inclusion Criteria

We included studies that used the MMAS-8 or the MGL Medication Adherence Scale questionnaires to assess the adherence levels to antihypertensive medications in hypertensive patients aged 60 years or older. The studies were also considered eligible for the review if they included patients younger than 60 years, but the adherence data for individuals aged ≥60 years were presented separately.

### Exclusion Criteria

We excluded studies published before 2000, using the MMAS-8 or the MGL Medication Adherence Scale scales on other than hypertensive patients treated with antihypertensive medications, intervention studies aimed at the improvement of medication adherence (unless the baseline adherence data were available), studies using maximum scores of the MMAS-8 (8 pts) or MGL Medication Adherence Scale (4 pts) to identify hypertensive patients as adherent, studies presenting absolute values of the MMAS-8 and the MGL Medication Adherence Scale scores rather than the percentages of adherent patients, as well as reviews, case reports and the studies we were unable to extract for full-text review.

### Review Process

**Figure 1:**
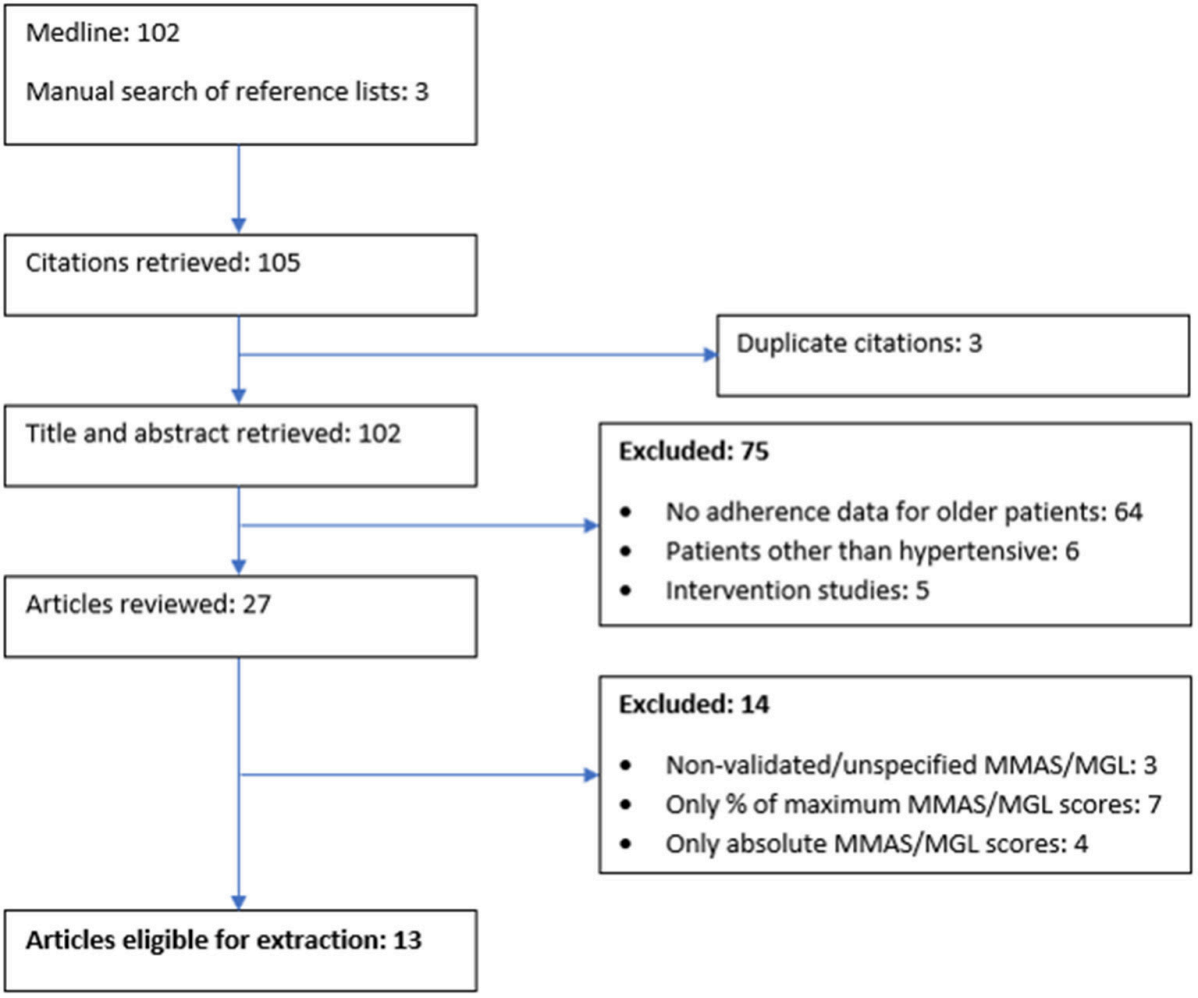
Flow diagram of the study.

During the first stage, all records were identified from searches of the electronic databases and duplicates were removed. During the second stage, two researchers (BU and IU) independently screened the titles and abstracts to identify the potentially eligible studies. During the third stage, studies that were potentially eligible were selected for full-text review (Figure 1). The disagreement was resolved by mutual consent after discussion.

### Data Extraction

The characteristics recorded for each study included authors names, publication year, country of origin, publication year and the year(s) the study was conducted, duration of data collection, sample size, study setting (hospital or community-based), questionnaire validity and the number and percentage of patients who are adherent and non-adherent to antihypertensive medication. The patients were considered adherent if they scored ≥6 pts. on the MMAS-8 scale or ≥3 pts. on the MGL Medication Adherence Scale. If available, also odds ratios (OR) with 95% confidence intervals (95% CI) for determinants of medication adherence were recorded.

### Analysis

The meta-analysis was conducted with Statistica 10 package (StatSoft, Tulsa, OK, United States). The Cochrane Q and the I^2^ were used to evaluate heterogeneity of studies. A random-effects model was used to combine studies showing heterogeneity of Cochrane Q *P* < 0.10 and I^2^ > 50 (Haidich, 2010). Strengthening the Reporting of Observational Studies in Epidemiology (STROBE) scale was used to assess the quality of the studies by categorizing them into high quality (≥75% of the STROBE checklist) and low quality (< 75% of the STROBE checklist) (von Elm et al., 2007). We performed subgroup analysis by MMAS type (MMAS-8 vs. MGL Medication Adherence Scale) and region (Western countries, i.e., Europe and the United States vs. other countries). Moreover, Egger and Begg tests representing funnel plots were used to assess the publication bias.

We did not conduct meta-analyses for the determinants of adherence, as adjusted ORs (aORs) were reported sporadically or were not comparable. Instead, we described the most common determinants, categorizing them into the five dimensions of adherence defined by the WHO: (1) social and economic factors, (2) health care team and system-related factors, (3) condition-related factors, (4) therapy-related factors, and (5) patient-related factors (WHO, 2003).

## Results

### Study Characteristics

Of 105 records initially identified, titles and abstracts of 102 studies were assessed for eligibility after exclusion of duplicates (Figure 1). A total of 89 studies were non-eligible for the meta-analysis because they included both patients ≥60 years of age and younger without specifying the adherence in the former group (*n* = 64), included other than hypertensive patients treated with antihypertensive medications (*n* = 6), analyzed the outcomes of interventions aimed at the improvement of medication adherence (*n* = 5), used non-validated or unspecified versions of the MMAS-8 or the MGL Medication Adherence Scale (*n* = 3), used maximum scores of the MMAS-8 or the MGL Medication Adherence Scale to identify hypertensive patients as adherent (*n* = 7), or presented only absolute values of the MMAS-8 and the MGL Medication Adherence Scale scores (*n* = 4). Eventually, 13 studies including a total of 5,247 hypertensive patients were available for the meta-analysis. Based on the percentages of satisfied STROBE criteria (von Elm et al., 2007), the quality of all studies was assessed as high or borderline high (Table 1).

**Table 1:** Study characteristics.

**References**	**Country**	**Study period**	**Study type**	**Setting**	***n***	**Age (yrs.)**	Tool^*^	**Cut-off value (pts)**	**Adherence (%)**	**STROBE criteria (%)**
Berni et al., 2011	Italy	Dec 2007–Jun 2009	Cross-sectional	Hospital	42	≥60	MMAS-8	≥6	60.0	79.5
Holt et al., 2013	United States	Aug 2006–Sep 2007	Cross-sectional	Community	1,075	≥75	MMAS-8	≥6	88.2	97.7
Muntner et al., 2013	United States	Aug 2006–Sep 2007	Cross-sectional	Community	1,391	≥65	MMAS-8	≥6	90.4	97.7
Lee et al., 2013	Hong Kong	Feb 2012–Apr 2012	Cross-sectional	Community	773	≥60	MMAS-8	≥6	72.3	93.2
Arshad, 2015	Pakistan	Aug 2014–Oct 2014	Cross-sectional	Community	45	>60	MGL	≥3	75.6	75.0
Kang et al., 2015	Hong Kong	Oct 2012–Mar 2013	Cross-sectional	Community	165	≥60	MMAS-8	≥6	58.5	95.5
Jankowska-Polańska et al., 2016	Poland	Jan 2015–Nov 2015	Cross-sectional	Community	296	≥60	MMAS-8	≥6	81.8	75.0
Haley et al., 2016	United States	Nov 2010–Mar 2013	Clinical trial	Community	243	≥75	MMAS-8	≥6	85.8	95.5
Hou et al., 2016	China	Sep 2013–Jun 2014	Cross-sectional	Community	585	≥60	MMAS-8	≥6	34.2	93.2
Okello et al., 2016	Uganda	Jan 2015–May 2015	Cross-sectional	Community	121	>60	MMAS-8	≥6	18.2	97.7
Teshome et al., 2017	Ethiopia	Mar 2015–May 2015	Cross-sectional	Hospital	141	>60	MGL	≥3	68.8	88.6
Jankowska-Polańska et al., 2017	Poland	Feb 2014–Apr 2015	Cross-sectional	Community	180	>65	MMAS-8	≥6	81.1	84.1
Al-Ruthia et al., 2017	United States	Aug 2013–Dec 2013	Cross-Sectional	Community	190	≥60	MMAS-8	≥6	78.9	79.5

*MMAS-8, Morisky Medication Adherence Scale; MGL, Morisky Green Levine Medication Adherence Scale; n, number of participants; yrs, years; pts, points; STROBE, Strengthening The Reporting of Observational Studies in Epidemiology*.

* *Use of the ©MMAS is protected by US copyright laws. Permission for use is required. A license agreement is available from: Donald E. Morisky, 294 Lindura Court, Las Vegas, NV 89138-4632; USA; dmorisky@gmail.com*.

Among the 13 eligible studies, there were 11 conducted with the MMAS-8 (Berni et al., 2011; Holt et al., 2013; Lee et al., 2013; Muntner et al., 2013; Kang et al., 2015; Haley et al., 2016; Hou et al., 2016; Jankowska-Polańska et al., 2016; Okello et al., 2016; Al-Ruthia et al., 2017) and two in which the MGL Medication Adherence Scale was used (Table 1) (Arshad, 2015; Teshome et al., 2017). Seven of those studies were conducted in Western countries: United States (*n* = 4) (Holt et al., 2013; Muntner et al., 2013; Haley et al., 2016; Al-Ruthia et al., 2017), Poland (*n* = 2) (Jankowska-Polańska et al., 2016, 2017), and Italy (*n* = 1) (Berni et al., 2011), and six in other countries: Hong Kong (*n* = 2) (Lee et al., 2013; Kang et al., 2015), China (*n* = 1) (Hou et al., 2016), Pakistan (*n* = 1) (Arshad, 2015), Ethiopia (*n* = 1) (Teshome et al., 2017), and Uganda (*n* = 1) (Okello et al., 2016).

All but one study (Haley et al., 2016) were cross-sectional analyses, and all but two (Berni et al., 2011; Teshome et al., 2017) were conducted in a community setting. The surveys were collected between August 2006 and November 2015. The sample sizes in the included studies ranged between 42 (Berni et al., 2011) and 1,391 (Muntner et al., 2013). Five studies included only older patients, with the cut-off values for age set at ≥60 years (Berni et al., 2011; Hou et al., 2016; Jankowska-Polańska et al., 2016; Al-Ruthia et al., 2017) or ≥65 years (Muntner et al., 2013). Mean age of hypertensive patients participating in those studies ranged from 68.4 ± 7.48 years (Hou et al., 2016) to 75.2 ± 5.7 years (Muntner et al., 2013). Among 2,504 participants of those studies there were 1,402 (56.0%) women and 1,102 (44.0%) men. Other eight studies (*n* = 2,743) involved both patients ≥60 years and younger, but their authors provided information about the percentages of adherence in persons aged ≥60 years (Lee et al., 2013; Kang et al., 2015), >60 years (Arshad, 2015; Okello et al., 2016; Teshome et al., 2017), >65 years Jankowska-Polańska et al., 2017, or ≥75 years (Holt et al., 2013; Haley et al., 2016). Unfortunately, we were unable to extract detailed demographic characteristics of patients ≥60 years who participated in those studies.

### Percentages of Adherence

**Figure 2:**
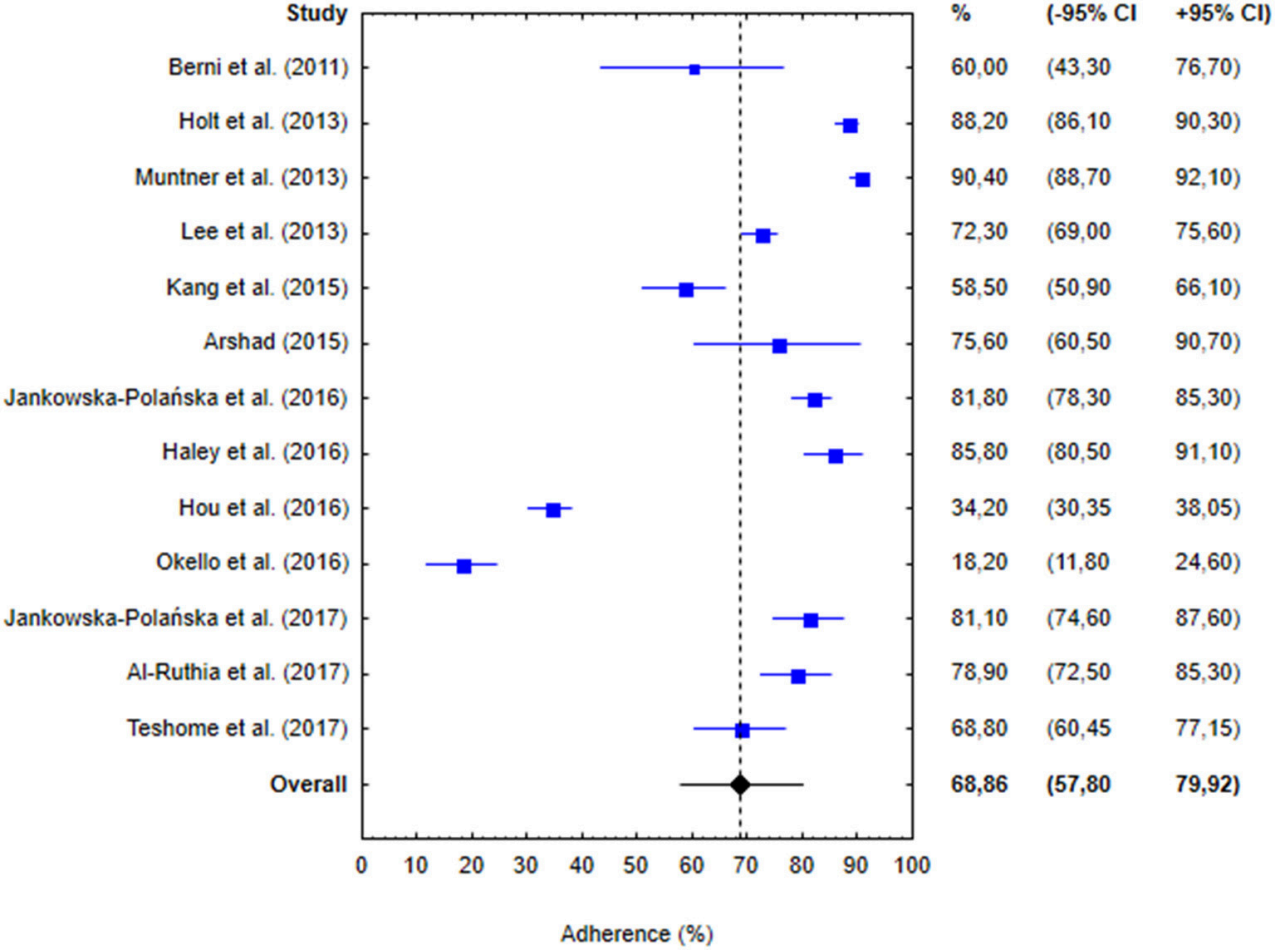
Percentages of hypertensive patients identified as adherent in 13 studies included in the meta-analysis, along with 95% confidence intervals (−95 to +95% CI). Blue boxes represent adherence in individual studies and blue whiskers correspond to 95% confidence intervals. Vertical dashed line and black diamond represent overall adherence determined in the meta-analysis and black whiskers correspond to 95% confidence interval.

The percentages of adherence reported in the studies included in the meta-analysis are presented in Figure 2. The proportions of adherent patients in individual studies varied from 18.2 (Okello et al., 2016) to 90.4% (Muntner et al., 2013), with the pooled percentage of adherence equal 68.86% (95% CI: 57.80–79.92%; Figure 2). Visual inspection of the forest plot and statistical test demonstrated considerable heterogeneity among the studies. Sensitivity analysis (each study sequentially excluded) revealed that the result of the meta-analysis was not dependent on the outcome of any of the individual studies (Table 2).

**Table 2:** *Post-hoc* sensitivity analysis for studies included in the meta-analysis (each study excluded).

**References**	**Adherence (%)**	**SE**	**(−95% CI**	**+95% CI)**	***p***	**Weight (%)**
Berni et al., 2011	69.50	5.85	58.04	80.97	<0.001	93.24
Holt et al., 2013	67.17	6.71	54.03	80.32	<0.001	92.03
Muntner et al., 2013	66.99	6.51	54.22	79.75	<0.001	92.02
Lee et al., 2013	68.55	6.26	56.29	80.82	<0.001	92.06
Kang et al., 2015	69.73	5.88	58.20	81.25	<0.001	92.30
Arshad, 2015	68.36	5.87	56.85	79.87	<0.001	93.04
Jankowska-Polańska et al., 2016	67.74	6.26	55.46	80.01	<0.001	92.07
Haley et al., 2016	67.41	6.03	55.59	79.24	<0.001	92.15
Hou et al., 2016	71.99	4.45	63.26	80.72	<0.001	92.08
Okello et al., 2016	73.20	5.00	63.40	83.00	<0.001	92.21
Jankowska-Polańska et al., 2017	67.83	5.99	56.09	79.56	<0.001	92.22
Al-Ruthia et al., 2017	68.01	5.99	56.27	79.75	<0.001	92.21
Teshome et al., 2017	68.87	5.92	57.25	80.48	<0.001	92.35
Overall effect	68.86	5.64	57.80	79.92	<0.001	100

*SE, standard error; CI, confidence interval; p, statistical significance p-value*.

**Figure 3:**
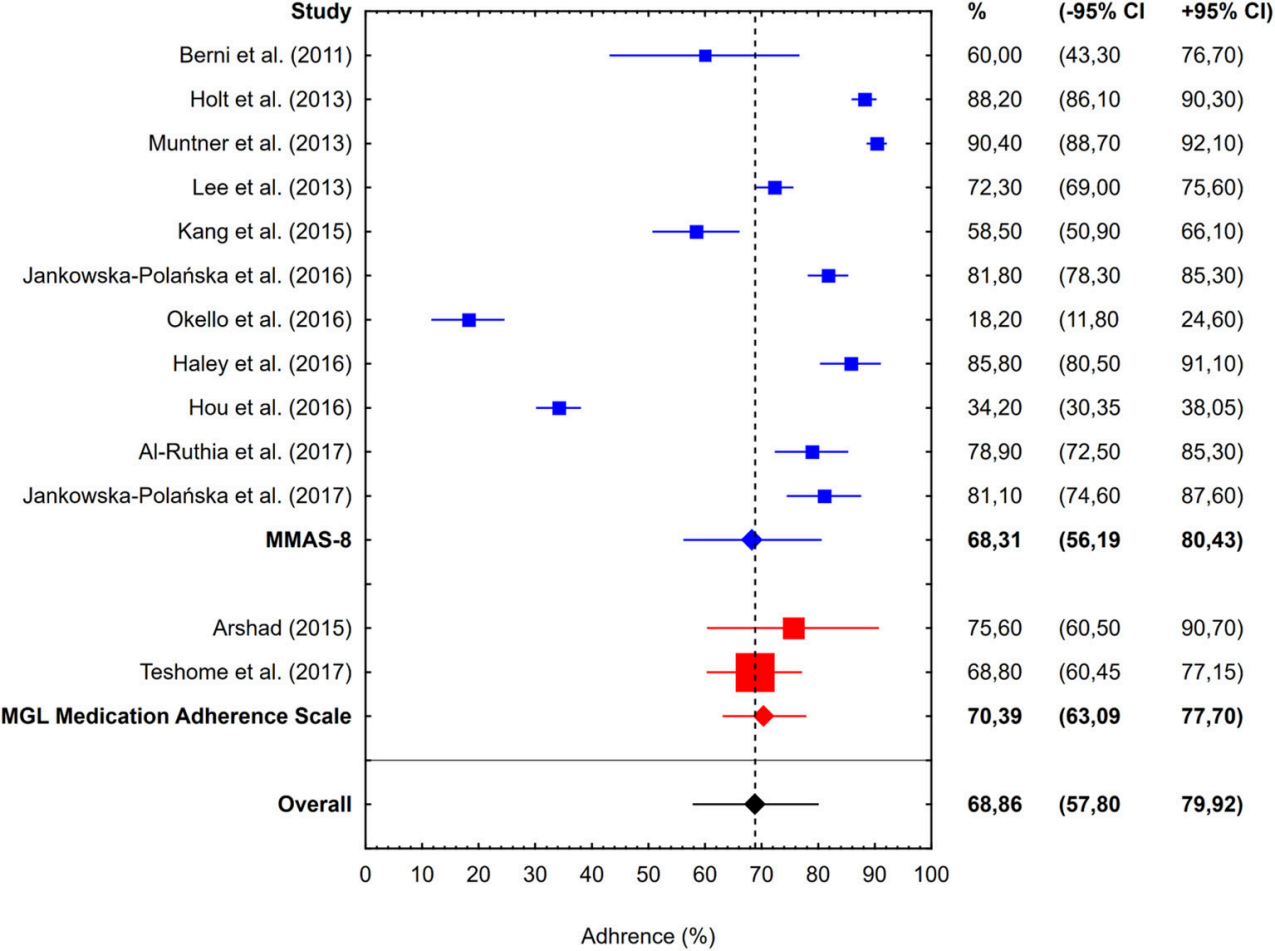
Percentages of hypertensive patients identified as adherent in 13 studies included in the meta-analysis, stratified according to the type of medication adherence scale used: MMAS-8 (11 studies, blue boxes, and whiskers) or MGL Medication Adherence Scale (2 studies, red boxes, and whiskers). Blue and red diamonds represent overall adherence determined in the studies using MMAS-8 and MGL Medication Adherence Scale, respectively. Meaning of other symbols identical as in Figure 2.

**Figure 4:**
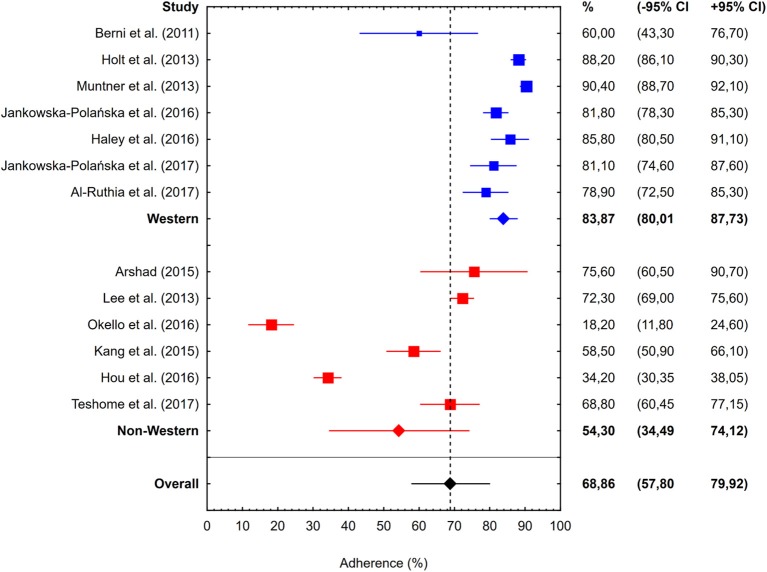
Percentages of hypertensive patients identified as adherent in 13 studies included in the meta-analysis, stratified according to the country of the study: Western (7 studies, blue boxes, and whiskers) or non-Western (6 studies, red boxes, and whiskers). Blue and red diamonds represent overall adherence determined in the studies involving patients from Western and non-Western countries, respectively. Meaning of other symbols identical as in Figure 2.

Subgroup analysis did not demonstrate a statistically significant difference in the percentages of adherent patients identified using the MMAS-8 and the MGL Medication Adherence Scales (68.31%, 95% CI: 56.19–80.43% vs. 70.39%, 95% CI: 63.09–77.70%, *P* = 0.773; Figure 3). However, the adherence among hypertensive patients from Western countries turned out to be significantly higher than in individuals from non-Western countries (83.87%, 95% CI: 80.01–87.73 vs. 54.30%, 95% CI: 34.49–74.12, *P* = 0.004; Figure 4).

### Publication Bias

Publication bias was not highlighted in the analysis, as confirmed by Egger and Begg tests representing funnel plots.

### Factors Affecting Adherence

A total of eight studies reported aORs for factors affecting adherence/non-adherence (Table 3). Factors included in more than one study were age, occupation, duration of hypertension, and a number of currently taken drugs. Some of the aORs represented the odds of being adherent and others of being non-adherent. To compare aORs from different studies, we transformed aORs for non-adherence into aORs for adherence, using the following formula: aOR(adherence) = 1/aOR(non-adherence).

**Table 3:** The five dimensions of factors affecting adherence.

**Dimensions**	**Factors presented in the articles**
Social and economic factors	Age (Lee et al., 2013; Kang et al., 2015), sex (Jankowska-Polańska et al., 2016), education (Jankowska-Polańska et al., 2017), occupation (Lee et al., 2013; Kang et al., 2015), place of residence (Teshome et al., 2017), concern about medical cost (Holt et al., 2013), payment mode (Okello et al., 2016)
Healthcare team and system-related factors	Availability of professional guidance (Holt et al., 2013)
Condition-related factors	Comorbidity (Arshad, 2015), blood pressure level control (Jankowska-Polańska et al., 2017), duration of hypertension (Lee et al., 2013; Jankowska-Polańska et al., 2017)
Therapy-related factors	Number of drugs taken currently (Jankowska-Polańska et al., 2017; Teshome et al., 2017)
Patient-related factors	Knowledge (Teshome et al., 2017), attitude (Holt et al., 2013), self-perceived health status (Kang et al., 2015), self-perception of aging (Hou et al., 2016)

Age, expressed as a continuous variable, was reported as a factor in two studies, with aOR equal 1.016 (95% CI: 1.001–1.032) (Lee et al., 2013) and 1.012 (95% CI: 1.002–1.022) (Kang et al., 2015) per each year of age, respectively. The same two studies reported occupation as a determinant of medication adherence; in the study conducted by Lee et al. (2013) the odds of being adherent were shown to be higher in unemployed or retired patients (aOR = 1.496, 95% CI: 1.017–2.200) than in employed ones, and Kang et al. (2015) demonstrated that employed persons were less likely to be adherent to antihypertensive medications (aOR = 0.782, 95% CI: 0.628–0.975). Two studies identified duration of hypertension as an independent determinant of medication adherence; according to both Lee et al. (2013) and Jankowska-Polańska et al. (2017), patients with more than a 10-year history of the disease were more likely to be adherent than those diagnosed with hypertension < 5 years earlier (aOR = 1.598, 95% CI: 1.115–2.291 and aOR = 1.93, 95% CI: 1.07–2.92, respectively). Finally, two studies demonstrated that the odds of being adherent were influenced by the number of currently taken drugs. Jankowska-Polańska et al. (2017) showed that individuals prescribed monotherapy (aOR = 1.67, 95% CI: 1.18–2.33) or one-tablet polytherapy (aOR = 3.70, 95% CI: 1.56–8.33) were more likely to be adherent than those receiving multiple drugs, and according to Teshome et al. (2017), the odds for adherence increased significantly in persons who received less than two antihypertensive agents per day compared with those treated with two or more medications (aOR = 3.04, 95% CI: 1.53–6.06).

## Discussion

In this meta-analysis, the percentage of patients aged ≥60 years who showed at least moderate adherence to antihypertensive treatment was estimated at 68.86% (95% CI: 57.80–79.92%), with the adherence rates in source studies varying considerably, from 18.2 (Okello et al., 2016) to 90.4% (Muntner et al., 2013). Adherence to antihypertensive treatment was a subject of many previous studies, but aside from a few included in this meta-analysis, none of them analyzed medication adherence in older persons. Pooled adherence rates documented in those studies were also highly heterogeneous, from 19 (Corrêa et al., 2016) to 87.7% (Hennein et al., 2018). Adherence to antihypertensive medications was also subjected to two meta-analyses. The adherence rates determined in those studies were lower than in our meta-analysis: 36.65 (Nielsen et al., 2017) and 54.8% (Abegaz et al., 2017). However, it needs to be stressed that none of those meta-analyses considered solely the studies of older patients. Furthermore, the meta-analysis published by Nielsen et al. (2017) included only the results of studies conducted in low- and middle-income countries.

Previous studies identified a plethora of factors that modulate medication adherence in hypertensive patients. In our meta-analysis, we found statistically significant differences in the adherence of patients from Western countries (Europe and United States) and other countries which to a large extent overlapped with the low- and middle-income countries analyzed by Nielsen et al. (2017). Potential causes of lower medication adherence in hypertensive patients from non-Western countries include socioeconomic factors, primarily financial and economic barriers in the access to healthcare services (Wong et al., 2010; Ambaw et al., 2012; Bhandari et al., 2015; Yue et al., 2015; Nielsen et al., 2017).

However, also the medication adherence of patients from high-income countries can be influenced by a number of factors. In this review, we identified four factors that exerted an independent effect on antihypertensive medication adherence in at least two studies: age, occupation, duration of hypertension and the number of currently taken drugs. Two studies included in our meta-analysis demonstrated that the odds of being adherent increased significantly per each year of age, by 1.2–1.6% (Lee et al., 2013; Kang et al., 2015). Those findings, as well as the fact that the pooled adherence determined in our study was higher than in two meta-analyses of younger patients (Abegaz et al., 2017; Nielsen et al., 2017), imply that seniors are more likely to be adherent to antihypertensive medications. However, the relationship between age and adherence is complex. The vast majority of previous studies, also those non-included in our meta-analysis (Degli Esposti et al., 2002; Krousel-Wood et al., 2004; Burnier, 2006; Hyre et al., 2007; Morisky et al., 2008; Carter and Foppe van Mil, 2010; Morisky and DiMatteo, 2011; Fernandez-Arias et al., 2014; Rajpura and Nayak, 2014; Akintunde and Akintunde, 2015; Alhaddad et al., 2016; Cummings et al., 2016; Li et al., 2016; Berlowitz et al., 2017; Khayyat et al., 2017) identified older age as a determinant of higher adherence. The authors of the previous studies postulated that older persons have more comorbidities, and thus, perceive themselves as sicker, which in turn makes them more adherent (Billups et al., 2000; Lee et al., 2013). In some studies, however, adherence to antihypertensive medications was shown to decrease with age (Jackevicius et al., 2002; Lam et al., 2007), probably as a consequence of age-related cognitive impairment, functional limitations, and problems with self-care. Hence, also those factors need to be considered when discussing medication adherence in older patients with hypertension.

Another factor determining medication adherence in two studies included in this systematic review was a broadly defined occupation of hypertensive patients. In the study conducted by Lee et al. (2013) the odds of being adherent were shown to be higher in unemployed or retired patients (aOR = 1.496) than in employed ones, and Kang et al. (2015) demonstrated that employed persons were less likely to be adherent to antihypertensive medications (aOR = 0.782). Higher adherence of retired patients with hypertension was also documented in another study that had been disqualified from present analysis (Li et al., 2016). According to Kang et al. (2015), adherence to medication schedule in employed patients might be partially compromised by their job duties. However, the evidence from the studies mentioned above, all conducted in China (Li et al., 2016) and Hong Kong (Lee et al., 2013; Kang et al., 2015), should be interpreted carefully, since in many countries, unemployed and retired persons have lesser access to healthcare services and limited financial resources, which might constitute a barrier to medication adherence (Zyczynski and Coyne, 2000).

In two studies included in this systematic review (Lee et al., 2013; Jankowska-Polańska et al., 2017), an independent predictor of higher adherence was a longer duration of hypertension, more than 10 years (aOR = 1.598 and aOR = 1.93, respectively). Similar observations were also reported by other authors whose studies were not included in this meta-analysis (Hyre et al., 2007; Yue et al., 2015; Mekonnen et al., 2017). Higher adherence among patients with a longer history of hypertension might be a consequence of better knowledge and experience with this condition, better patient-physician relationships and greater trust in physician's advice (Svensson et al., 2000; Lee et al., 2013; Mekonnen et al., 2017).

Finally, two studies demonstrated that the adherence to antihypertensive treatment increased inversely to the number of currently taken drugs. Jankowska-Polańska et al. (2017) showed that individuals prescribed monotherapy or one-tablet polytherapy were more likely to be adherent than those receiving multiple drugs (aOR = 1.67 and aOR = 3.70, respectively), and according to Teshome et al. (2017), the odds for adherence increased significantly in persons who received less than two antihypertensive agents per day, as compared with those treated with two or more medications (aOR = 3.04). Also, other authors demonstrated that patients were more adherent to antihypertensive therapies if they were prescribed only one drug or a single daily dose (Dunbar et al., 2008; Kamran et al., 2014), or found an inverse correlation between the number of prescribed drugs and the adherence (Fernandez-Arias et al., 2014; Akintunde and Akintunde, 2015; Mroczek et al., 2015; Napolitano et al., 2016).

Aside from the factors mentioned above, representing five dimensions of adherence defined by the WHO (2003), medication adherence may also be influenced by the method used to determine this parameter. While the authors of both previously mentioned meta-analyses (Abegaz et al., 2017; Nielsen et al., 2017) excluded the studies using an older four-item version of the MGL Medication Adherence Scale, our study demonstrated that the adherence estimates obtained with this scale did not necessarily differ significantly from those determined with the MMAS-8. However, contrary to Nielsen et al. (2017), we excluded studies whose authors used maximum scores of the MMAS-8 (8 pts.) or the MGL Medication Adherence Scale (4 pts.) to identify hypertensive patients as adherent, since the subgroup analysis (not shown) demonstrated that inclusion of those studies led to a considerable underestimation of the adherence rates.

### Potential Limitations

This study might suffer from some potential limitations. First, our search was limited only to publications available in the PubMed, Scopus, and Google Scholar, and to English-language articles; hence, it might not cover all studies dealing with the problem in question. Second, the number of eligible studies was quite small (*n* = 13) and thus, our meta-analysis might have been underpowered. Third, older hypertensive patients were defined using various cut-off values for age (≥60, >60, >65, and ≥75 years), which might constitute a source of bias. Fourth, we could not exclude that at least some of the patients participating in the analyzed studies suffered from comorbidities which might have affect their medication adherence, e.g., diabetes mellitus or elevated serum lipids. Fifth, none of the studies analyzing potential determinants of medication adherence included solely the older patients, so it is unclear whether their results can be extrapolated onto this age group. Sixth, we considered solely the studies based on the two medication adherence scales, MMAS-8 and MGL. Hence, it cannot be excluded that the results obtained with other self-reported scales would be slightly different. However, preliminary literature search demonstrated that MMAS-8 and MGL were used in the vast majority of studies dealing with the problem in question, and only few authors analyzed adherence with other instruments, e.g., Hill-Bone Compliance Scale. We did not include those studies in our meta-analysis to avoid a potential confounding effect of different adherence scale.

## Conclusion

In summary, results from our meta-analysis indicated that medication adherence in the oldest old hypertensive patients seems to be higher than in younger persons. Adherence in older persons was associated with age, socioeconomic status and therapy-related factors.

## Ethics Statement

This study was carried out in strict accordance with the recommendations in the STROBE and PRISMA guidelines. Ethics committee or institutional review board permission is not required for conducting a systematic review and meta-analysis.

## Author Contributions

BU, EJ, and IU, were responsible for the conception and design, acquisition of data, analysis, interpretation of data, and wrote this manuscript. BU, EJ, IU, and DM were responsible for drafting the initial manuscript and revising it critically for important intellectual content. DM improved the grammar, syntax, and flow of our manuscripts prior to submission. All authors read and approved the final manuscript.

### Conflict of Interest Statement

DM is the developer/owner of the MMAS and received funds for issuing licenses sought by health-care entities for their use. He was not involved in the analysis of the data. The remaining authors declare that the research was conducted in the absence of any commercial or financial relationships that could be construed as a potential conflict of interest.
